# Strategies to evaluate outcomes in long-COVID-19 and post-COVID survivors

**DOI:** 10.1186/s13027-021-00401-3

**Published:** 2021-10-30

**Authors:** Anna Crispo, Sabrina Bimonte, Giuseppe Porciello, Cira Antonietta Forte, Gaia Cuomo, Concetta Montagnese, Melania Prete, Maria Grimaldi, Egidio Celentano, Alfonso Amore, Elvio de Blasio, Francesca Pentimalli, Antonio Giordano, Gerardo Botti, Giovanni Baglio, Pierpaolo Sileri, Marco Cascella, Arturo Cuomo

**Affiliations:** 1 Epidemiology and Biostatistics Unit, Istituto Nazionale Tumori-IRCCS-Fondazione G. Pascale, 80131 Naples, Italy; 2 Division of Anesthesia and Pain Medicine, Istituto Nazionale dei Tumori Fondazione G. Pascale, 80131 Naples, Italy; 3 SSD Chirurgia Melanoma E Dei Tumori Cutanei, Istituto Nazionale Tumori-IRCCS-Fondazione G. Pascale, 80131 Naples, Italy; 4 Multidisciplinary Emergency Unit for COVID-19 Campania, 80100 Naples, Italy; 5 Cell Biology and Biotherapy Unit, Istituto Nazionale Tumori-IRCCS-Fondazione G. Pascale, 80131 Naples, Italy; 6 Ministry of Health, 00153 Rome, Italy; 7 Center for Biotechnology, College of Science and Technology, Sbarro Institute for Cancer Research and Molecular Medicine, Temple University, Philadelphia, PA 19122 USA; 8 Scientific Direction, Istituto Nazionale Tumori IRCCS Fondazione G. Pascale, Naples, Italy; 9 Head of the Unit “Research and International Relations”, Italian National Agency for Regional Health Services - AGENAS, 00187 Rome, Italy; 10 Università Vita Salute San Raffaele, 20132 Milan, Italy

**Keywords:** COVID-19 pandemic, Public health, Neurocognitive disorders, Intensive care units

## Abstract

SARS-CoV-2 infection can impact the physical, cognitive, mental health of patients, especially in those recovered in intensive care units. Moreover, it was proved that the effects of the virus may persist for weeks or months. The term long-COVID or post-COVID syndrome is commonly used for indicating a variety of physical and psychological symptoms that continue after the resolution of the acute phase. This narrative review is aimed at providing an updated overview of the impact of physical, cognitive, and psychological health disorders in COVID-19 survivors, by summarizing the data already published in literature in the last year. Studies cited were found through PubMed searches. We also presented an overview of the post-COVID-19 health consequences on three important aspects: nutritional status, neurological disorders, and physical health. Moreover, to activate a correct health planning policy, a multidisciplinary approach for addressing the post- COVID-19 issue, has been proposed. Finally, the involvement of health professionals is necessary even after the pandemic, to reduce expected post-pandemic psychosocial responses and mental health disorders.

## Introduction

Clinical manifestations of COVID-19 vary from asymptomatic forms to self-limiting conditions, up to severe manifestations featuring respiratory and multi-organ involvement [1–6]. Epidemiological data reveal that up to 20% of COVID-19 patients progress to a severe condition that requires hospitalization [7]. Among those who are hospitalized, up to one-quarter need intensive care unit (ICU) admission, making them more vulnerable to secondary pneumonia, cardiac injury, sepsis, kidney injury, and neurologic disorders [8].

The term ‘Long COVID’ or ‘Post-COVID’ is commonly used to describe an array of signs and symptoms that are present after acute COVID-19. The UK’s National Institute for Health and Care Excellence described the Long COVID as “ongoing symptomatic COVID-19” (symptoms between 4 and 12 weeks) and “Post-COVID syndrome” when symptoms lasting longer than 12 weeks [9]. Recently the Long COVID has been recognized by the World Health Organization (WHO) as an international healthcare concern and an “emergency-use” ICD code has been issued [10]. The study of the long-term outcome of patients discharged from ICU revealed significant disabilities, collectively known as post-intensive care syndrome (PICS) [2], affecting physical, cognitive, and psychological health. PICS includes symptoms like generalized weakness, memory disturbances, poor concentration, depression, anxiety, and post-traumatic distress disorder (PTSD) [2].

PICS incidence can be quite high: affecting up to 60% of the patients for what concerns cognitive and psychological symptoms and between 25 and 60% for what concerns neuromuscular disorders [11–13]. Furthermore, the PICS can last many years, affecting the health-related quality of life (HR-QoL) and the ability to return to work [14, 15]. Patients who survive acute distress respiratory syndrome (ARDS) could develop chronic pain and it would be possible that patients with a severe type of COVID-19 disease could develop similar complications [16]. Moreover, the psychological burden of ICU admission and stay of a patient can affect also his/her relatives who can develop symptoms of PTSD as well [17].

Here, we reviewed the clinical studies on neurocognitive disorders in Post-COVID patients. Studies cited in this narrative review were discovered through PubMed searches. PubMed was searched for clinical articles published in the last two years related to neurocognitive disorders, covid-19 survivors, physical cognitive, and mental health disorders. Based on these data, we proposed an overview of the COVID-19 health consequences by focusing on three important aspects: neurological disorders, physical health, and nutritional status.

## The post-COVID syndrome

The Post-COVID Syndrome includes persistent symptoms related to residual inflammation, organ damage, non-specific effects from the hospitalization post-intensive care syndrome, social isolation, or impact on pre-existing health conditions [18–20].

Post-COVID syndrome could be due to various mechanisms such as post-ICU syndrome, post-viral fatigue syndrome, permanent organ damage, or others [21]. Even if Long COVID was initially thought to be limited to survivors of hospital care and to those admitted to the ICU, it is now evident that most cases are described even in those who were not hospitalized or who did not immediately seek medical care [22–24].

The most frequent alterations include headache, dizziness, balance and coordination disorders, difficulty in attention, concentration, and memory, as well as chronic fatigue, insomnia, changes in taste and smell, depression, and anxiety. These physical, psychological, and neurocognitive symptoms are close to those present in post-traumatic stress disorder (PTSD) [25, 26].

Several studies observed persistent symptoms and unexpected substantial organ dysfunction after SARS-CoV-2 infection in an increasing number of patients after recovering from their initial illness [27–29].

Data from a prospective cohort study on 270 COVID-19 survivors confirmed a post-COVID-19 syndrome in half of the patients experiencing symptoms such as fatigue and respiratory (dyspnea) or neurological complaints, 10–14 weeks after disease onset [30, 31] showed that 76% of hospitalized COVID-19 survivors reported at least one symptom that persisted, with fatigue or muscle weakness being the most frequently reported symptom, 6 months after illness onset.

Among ICU COVID-19 survivors several patients face impairments regarding their cognitive and mental health or physical function far beyond their hospital discharge [32].

Data from a UK study on the post-discharge impact of COVID‐19 infection on the health status of 100 survivors (32 ICU) revealed that: fatigue was the most common reported symptom in both ICU COVID-19 survivors (72%) and COVID-19 survivors (60%), followed by breathlessness (66% and 43%, respectively) and psychological distress (47% and 24%, respectively). Moreover, data showed a clinically significant HR-QoL in both ICU COVID-19 survivors (69%) and COVID-19 survivors (46%). Sixty percent of the ICU COVID-19 survivors and 15% of the COVID-19 survivors remained off‐sick from work after 4 weeks or more since discharge [29].

Data collected among symptomatic adults tested in outpatient settings (patients with mild COVID-19 without hospitalization) reported that 94% experienced one or more symptoms (cough 43%, fatigue 35%, or shortness of breath 29%) after infection onset, resulting in prolonged illness [28]. Persistent symptoms (such as anosmia/ageusia, dyspnea, or asthenia) have been reported in two-thirds of patients with non-critical COVID-19 [30]. The ongoing COVID-19 pandemic and the occurrence of Post-COVID syndrome has highlighted the PICS issue and the complex rehabilitation needs for people with severe illness and long ICU stays as well as for COVID-19 survivors that have not been hospitalized. The persistence of various symptoms in Long-COVID patients is a major health issue worldwide. Monitoring and treatment of patients with post-COVID syndrome are necessary to ensure rehabilitation and recovery of general functions. These findings support the need for a multidisciplinary approach to the care of this vulnerable population and to conduct research studies during 1–2 years of follow-up, as is currently happening in the UK and USA [31, 33, 34] (see Table 1).

**Table 1:** Studies of Physical health, Neurological disorders, and Nutritional status during the COVID-19 pandemic

References	Design	Country	Sample size (N)	Male (%)	Mean age (years)	Categories (health care workers, patient, hospitalized/ICU, general public)	Physical health	Neurological disorders	Nutritional status	COVID-19 status	Main findings
							N (%)	N (%)	N (%)	N (%)	
[90]	Cross-sectional study	UK	431.051	55.8	57.2	Hospitalized	n.a	psychological distress, neuroticism	n.a	908 hospitalized for COVID-19	Association of lower cognitive function based on a test of verbal and numerical reasoning with a higher risk of COVID-19
[91]	Observational study	Italy	50	n.a		Hospitalized	DEPENDENCE for motor or respiratory functions	n.a	(90%) dysphagia and need of a modified diet’s consistency or nasogastric feeding (45%) high risk and (26%) moderate risk of malnutrition BMI improvement 14 (43.7%)	50 (100%) positive to COVID-19	three-step nutritional protocol to ensure optimal nutritional status and improve clinical outcomes in COVID19 patients in a rehabilitation unit
[92]	Web-based cross-sectional survey	Italy	2291	25.3	30.0	General public	n.a	poor sleep quality (57.1%), high anxiety (32.1%), high distress (41.8%), PTSD symptomatology (7.6%)	n.a	Positive to COVID-19 9 (0.4%)	Correlation between epidemic with anxiety, sleep disorders and PTSD
[93]	Multicenter study	India	906	35.7	29	Healthcare workers	Headache 289 (31.9%), Throat pain 304 (33.6%), no symptoms 302 (33.3%)	moderate to very-severe depression 48 (5.3%), moderate to extremely severe anxiety 79 (8.7%), moderate to extremely severe stress 20 (2.2%), and moderate to severe levels of psychological distress 34 (3.8%), Lethargy 241(26.6%), Insomnia 190(21.0%)	n.a	n.a	significant association between the prevalence of physical symptoms and psychological outcomes among healthcare workers during the COVID-19 outbreak
[94]	Web-based cross-sectional survey	Italy	2291	25.3	30.0	General public	n.a	Higher risk of psychopathological symptoms in females (OR 2,32) younger than 50 years (OR > 1,68). Higher risk of developing anxiety was higher in females (OR = 3,10 younger than 50 (OR > 1,47) and undergraduates (OR = 1,68) Higher risk of PTSD symptomatology associated with female (OR 2,39)	n.a	9 (0.4%) positive to COVID-19	COVID-19 pandemic can be related to anxiety, changes in mood, high psychopathological symptomatology, and could be associated with the development of PTSD
[95]	Web-based cross-sectional survey	China	7236	45.4	35.3	Healthcare workers, General public	n.a	Generalized anxiety disorder, depressive symptoms and was significantly higher in general public, Healthcare workers reported highest rate of poor sleep quality. Generalized anxiety disorder 2540 (35.1%), depressive symptoms 1454 (20.1%), poor sleep quality 1317 (18.2%)	n.a	n.a	Major mental health burden during COVID outbreak
[96]	Online survey	Canada	1908	19.6	42	n.a	40.5% of inactive and 22,4% of active individual became less active 33% of inactive and 40.3% of active became more active 28.3% of inactive altered their type of activity, 39.6% of active maintained their type	Inactive scored significantly lower on the mental health assessment than the active participants, however there is no significant difference in generalized anxiety.; inactive participants that were more active indicated higher levels of social, emotional and psychological health, and lower levels of anxiety; inactive with mild anxiety were more physically active than participants with moderate anxiety; inactive participants with severe anxiety spent fewer minutes in outdoor physical activity than individuals with low or mild anxiety	n.a	n.a	Strong association between physical activity and well-being;
[3]	Observational study	Italy	143	62.9	56.5	Hospitalized	Post covid-19: 53.1% of individuals showed fatigue, 43.4% dyspnoea, 27.3% joint pain, 21.7% chest pain, Fatigue 53%	n.a	n.a	143 (100%) patient’s post-recovery from COV19	This study found that in patients who had recovered from COVID-19, 87.4% reported persistence of at least 1 symptom, particularly fatigue and dyspnea
[97]	Web-based cross-sectional survey	China	994	14.5	Aged 25– 40 years (63.4%)	Healthcare workers	n.a	(36.9%) subthreshold mental health disturbances, (34.4%) mild disturbances, (22.4%) moderate disturbances, and (6.2%) had severe disturbance	n.a	None	These findings emphasize the importance of being prepared to support frontline workers through mental health interventions at times of widespread crisis
[98]	Web-based cross-sectional survey	China	9684	23.3	813 (64.7%) were aged 26–40 y	Healthcare workers	67 (60.9%) had fever, 66 (60.0%) myalgia or fatigue, 62 (56.4%) cough, 55 (50%) sore throat, 50 (45.5%) muscle ache	634 (50.4%) depression,560 (44.6%) anxiety, 427(34%) insomnia, 899 (71.5%) distress	n.a	110 (1%) positive to COVID-19	These findings suggest that front-line healthcare workers exposed to COVID-19 have a high risk of developing unfavorable mental health outcomes and may need psychological support
[99]	Observational study	India	470	31.7	31	Healthcare workers, General public	n.a	60 (14.5%) participants showed anxiety, 42 (8.9%) depression, 31 (6.6%) stress, 36 (7.7%) post-traumatic stress disorder Anxiety was higher among nonmedical health care workers than medical personnel (20.7% versus 10.8%; adjusted prevalence ratio, 1.85 [95% CI, 1.15–2.99]; *P* = 0.011) Higher mean DASS-21 anxiety and stress subscale scores and higher IES-R total and subscale scores were observed in nonmedical health care workers	n.a	None	Nonmedical health care personnel are at highest risk for psychological distress during the COVID-19 outbreak. Early psychological interventions targeting this vulnerable group may be beneficial
[100]	Online survey	UK	153	48	71	General public	n.a	39 (31%) presented altered mental status, comprising 9 (23%) patients with unspecified encephalopathy and 7 (18%) encephalitis. 23 (59%) with altered mental status fulfilled the clinical case definitions for psychiatric diagnoses as classified by the notifying psychiatrist or neuropsychiatrist, and 21 (92%) of these were new diagnoses 6 (26%) had a neurocognitive (dementia-like) syndrome, and four (17%) had an affective disorder	n.a	114 (92%) confirmed 5 (4%) probable 5 (4%) possible	This study identified acute presentations of new-onset complications of COVID-19. Ischemic stroke was common in our cohort
[101]	Observational study	China	1738	Survey 1: 32.7; Survey 2: 25	Survey 1: 21,4–30,8y (53,1%); Survey 2: 21,4–30,8y (46,5%)	General public	n.a,	Measured by DASS-21 Mean score (SD), survey 1: 7,76 (7,74) stress, 6,16 (6,57) anxiety, 6,25 (7,16) depression; survey 2: 7,86 (7,93) stress, 6,15 (6,94) anxiety, 6,38 (7,39) depression. NS comparison between surveys	n.a	n.a	Protective factors included high level of confidence in doctors, perceived survival likelihood and low risk of contracting COVID-19, satisfaction with health information, personal precautionary measures. Governments should focus on effective methods of disseminating unbiased COVID-19 knowledge, correct containment methods, availability of essential services/commodities, and sufficient financial support
[102]	Web-based cross-sectional survey	China	2182	35.8	18-60y, n = 2,101 (96,3%)	Healthcare workers, General public	n.a	Medical health workers showed higher prevalence rates of insomnia (38.4 vs. 30.5%, p < 0.01), anxiety (13.0 vs. 8.5%, p < 0.01), depression (12.2 vs. 9.5%; p = 0.04), somatization (1.6 vs. 0.4%; p < 0.01), and obsessive–compulsive symptoms (5.3 vs. 2.2%; p < 0.01) than nonmedical health workers	n.a	n.a	During the COVID-19 outbreak, medical health workers had psychosocial problems and risk factors for developing them. They needed attention and recovery programs
[46]	Observation study	China	214	40.7	52.7	Hospitalized	Compared with patients with non-severe infection, patients with more severe infection had skeletal muscle injury 17 (19.3%) vs 6 (4.8%)	78 patients (36.4%) had neurologic manifestations: Compared to patients with non-severe infection, patients with more severe infection had neurologic manifestations, such as acute cerebrovascular diseases 5 (5.7%) vs 1 (0.8%), impaired consciousness 13 (14.8%) vs 3 (2.4%)	n.a	214 (100%) positive to COVID-19	During the pandemic, patients with neurologic manifestations should have SARS-CoV2 infection suspected, to avoid delayed diagnosis or misdiagnosis and to lose the ability to treat and prevent transmission
[56]	Observational study	China	201	63.7	51	Hospitalized	188 (93.5%) had Fever, 163 (81.1%) cough, 80 (39.8%) dyspnea, and 65 (32.3%) had fatigue or myalgia 66 (32.8%) had fever with fatigue, myalgia, or headache;	7 (3.5%) had nervous system disease	n.a	201 (100%) positive to COVID-19	Older age was associated with a high risk of developing ARDS and death: this may have been due to a weaker immune response from this category
[57]	Editorial	Europe	n.a	n.a	n.a	n.a	n.a	n.a	COVID-19 patient may have altered nutritional status characterized by malnutrition and body weight loss	n.a	The nutritional approach in SARS-CoV-2 patients in the ICU, internal medicine ward and general health care should not be underestimated, and dietary intervention should be an important part of the care provided to these patients..
[54]	Observational study	Denmark	498,151	32,775 (6.5%)	43	Hospitalized and non- hospitalized	Increased risk of receiving hospital diagnoses of dyspnoea and venous thromboembolism for SARS-CoV-2-positive individuals compared with negative individuals	No increased risk of serious complications of SARS-CoV-2 infection, ischaemic stroke, encephalitis, psychoses	n.a	1310 positive to COVID-19	For those with SARS-CoV-2 who do not require hospitalization, the risk of having serious complications such as venous thromboembolism, ischaemic stroke, and psychoses is low

### Potential tools to evaluate outcomes in long-COVID and post-COVID patients: our proposal

Cognitive, physical, and psychological dysfunction reported by COVID19 patients can have profound effects on the HR-QoL [32].

We proposed a multimodal process as well as the sequence of several aspects of the health-related quality of life (HRQoL) contributing to the impact of the disease on cognitive, physical, and nutritional outcomes by considering the set of the following tools:

1. The Short Form Health Survey 36 (SF-36), which is a short questionnaire (36 items) that evaluates eight dimensions: physical functioning (10 items), social functioning (2 items), limitations due to physical problems (4 items), limitations due to emotional problems (3 items), mental health (5 items), energy/vitality (4 items), pain (2 items) and perception of general health (6 items) [35]. The SF-36 investigates the health changes of an individual compared to the previous year. As the tool evaluates the state of health in general, it is suitable for studies in the general population and transversal or longitudinal investigations on specific diseases, and treatments. Due to its characteristic of a general questionnaire, it needs to be accompanied by specific questionnaires when studying patient populations.
2. The Barthel Index, developed to measure improvements in individuals with a chronic disability who underwent rehabilitation programs, is commonly used in post-ARDS patients [36].
3. The Psychological General Well-Being Index (PGWBI) is designed for providing an index for measuring subjective well-being or suffering. It is composed of 22 items to assess anxiety, depression, positive well-being, self-control, general health, and vitality[37].
4. The EuroQoL [38]. It represents the attempt to develop a standardized, general tool for describing and evaluating HRQoL regardless of the specific disease. It is a questionnaire consisting of five dimensions and an analog self-assessment scale.
5. The Pittsburgh Sleep Quality Index (PSQI), for assessing sleep quality[39].
6. The Mini-Mental Test investigates the neurocognitive and functional state through simple targeted questions as well as small graphical tasks. It explores different domains of brain function, such as orientation, memory, attention and calculation, the ability to recall certain acquisitions, language, etc. [40].
7. The Brief Pain Inventory (BPI) rapidly assesses the severity of pain and its impact on functioning [41].
8. PTSS-14 (Post Traumatic Stress Syndrome 14 items) is a screening instrument to identify the patients who developed the Syndrome [42].
9. HADS (Hospital Anxiety and Depression Scale) to evaluate the level of depression and anxiety of the patients discharged at home [43].
10. MNA (Mini Nutritional Assessment) a nutritional educational program to assess nutritional status in patients in healthcare settings, appears ideal for patients with COVID -19, alongside a clinical and Para clinical evaluation [44, 45].

### Focus on three aspects to manage COVID-19 survivors

We underline three important aspects to manage COVID-19 survivors: (1) neurological disorders, (2) physical health, (3) nutritional status.

(1) Neurological disorders

Accumulated pieces of evidence highlighted that SARS-CoV-2 affected the nervous system [46–48]. In patients with a severe form of the disease, neurological manifestations were more evident. As reported by Mao et al. [46], neurologic manifestations affected or the central nervous system (CNS) (dizziness, headache, impaired consciousness, acute cerebrovascular disease, ataxia, and seizure), or the peripheral nervous system (PNS) (nerve pain and impairment of vision, test, and smell) or the skeletal muscular apparatus (injury). Among CNS alterations, the acute cerebrovascular disease was more evident in older patients and with severe infection and included cerebral hemorrhage and ischemic stroke diagnosed by clinical symptoms and head CT. Carfi et al. [3] demonstrated that worsened quality of life was observed in 44.1% of patients, and 87.4% reported persistence of at least 1 symptom, particularly fatigue and dyspnoea. The pathologic mechanism underlying the CNS invasion of SARS-CoV-2 is presumably like that of other respiratory viruses. Specifically, SARS-COV-2 can invade the CNS through the hematogenous or retrograde neuronal route. Since SARS-CoV-2 infects a large part of the world's population, understanding the potential neurologic implications of COVID-19 will help clinicians to identify and intervene in neurologic morbidity during and after the pandemic.

(2) Physical health

Patients who have undergone intensive care after discharge may experience a post-intensive care syndrome (PICS) characterized by physical, mental, cognitive [1], and nutritional problems [49]. The impact of ICU on physical function can impair daily activities, involving the neuromuscular, cardio-respiratory, and skeletal systems: these individuals very often report inability to return to work, musculoskeletal weakness and difficulty walking, impaired lung and respiratory function [50–53].

Most of the complications due to COVID-19 described were related to hospitalized patients and therefore not associated with patients who received home care. Few studies have evaluated the presence of complications in patients positive for SARS-Cov-2 who did not require hospital care but were still positive for SARS-Cov-2. In a population-based cohort study in Denmark, it was found that the risk of severe complications after COVID-19 in non-hospitalized patients about 6 months after the infection is very low, but these still have a higher risk for venous thrombotic events than people without disease and negative for SARS-Cov-2 [54]. There are currently no other studies evaluating the long-term effects of the virus in non-hospitalized patients beyond six months of infection.

(3) Nutritional status

Previous studies have highlighted the poor nutritional status of patients upon admission and during their stay in intensive care. The greater propensity for malnutrition and wasting is more visible in these critically ill patients, due to their developed metabolic disorders [49]. Nutritional status has long been considered an important factor that can influence the outcome of various infectious diseases, including viral pneumonia caused by SARS-CoV2 (COVID-19) [55, 56]. In COVID-19 patients, an altered nutritional status characterized by malnutrition and loss of body weight can be found and due to various causes [57, 58], including dyspnoea, anorexia, dysphagia, nausea, vomiting, diarrhea, increased energy requirements [59] advanced age, frailty, comorbidity [57] and prolonged hospital stay in ICU [58]. Currently, there are no specific dietary guidelines for post-COVID-19 patients with PICS disorders. However, several aspects could be considered to improve the impairments of cognitive functions. Eating habits can affect cognitive abilities [60]: unbalanced diets can have an overall negative impact on cognitive and mental health [61–63], negatively affecting the ability to reason, attention, and memory [61, 62, 64] and promote dementia and depression [65–68]. Greater adherence to a diet that includes healthy foods such as vegetables, fruits, seafood, lean meats, and whole grains, reduces the likelihood of suffering from depression or anxiety [69–71]. Nutrients such as vitamins (B1, B6, B12, B9, C, E, D), polyphenols, ω-3 fatty acids, minerals (iron, zinc, selenium), and foods with a low glycemic index have inhibitory action against oxidative stress and neuroinflammation [72, 73], and positively influence cognitive function [68, 74–84]. In this regard, several studies have reported that greater adherence to the Mediterranean diet was associated with an improvement in cognitive function and a reduced risk of cognitive impairment [83, 85–89]. It would be useful to monitor body composition, using methods such as bio impedance-analysis (BIA) or plicometry, and the nutritional status using the MNA [44, 45], to offer the most adequate nutritional support that contributes to reduce physical and cognitive complications both in post-hospitalization and in the long term.

## Conclusions

**Fig. 1:**
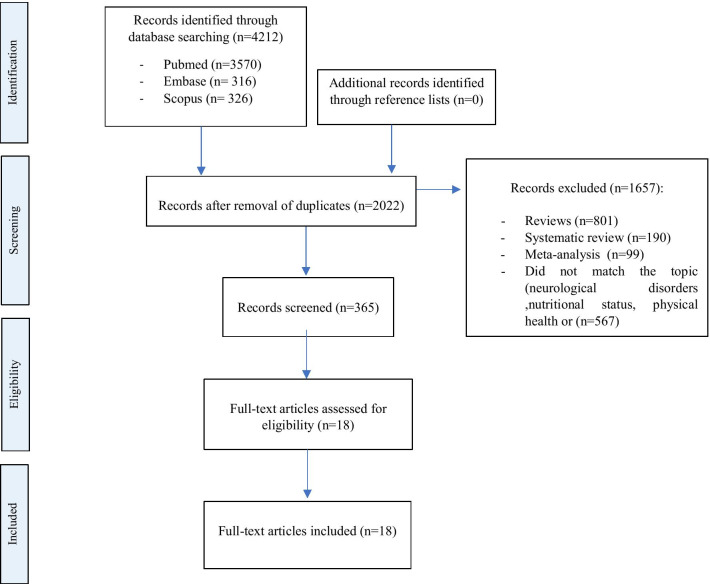
PRISMA flow diagram

The Sars-Cov-2 is an invisible enemy that makes us feel constantly under threat, it can infect people at any time, and this can generate different responses in subjects: anxiety, depression, panic, sleep, concentration disorders, and fatigue. All normal and legitimate reactions, however, must be contained to try to limit the effects, allowing us to better face the emergency we are experiencing. COVID-19 survivors, after clinical recovery, may have neurocognitive damage that should not be underestimated, and the extent and duration of which is not yet known. As we reported, also the COVID-19 survivors (without hospitalization), reported the post- COVID-19 syndrome. The most frequent alterations found are headache, balance and coordination disorders, difficulty in attention, insomnia, changes in taste and smell, depression, anxiety, physical and nutritional dysfunctions. The isolation, the hospitalization, the drama of the health emergency could have been decisive in the onset of some of these symptoms. Overall, the impact of post- COVID-19 syndrome should be considered as the potential cause of a delayed pandemic that may have a major public health impact in the medium to long term. Thus, preventive interventional approaches mitigating social impact should be considered as an integral part of the response to the crisis during pandemics. Moreover, the involvement of specific health professional figures is needed even after the pandemic, to manage and care for an increased number of patients (Fig. 1).

## Data Availability

Data sharing not applicable to this article as no datasets were generated or analyzed during the current study.

## Abbreviations

PICS: Post-intensive care syndrome
ICUs: Intensive care units
PTSD: Post-traumatic distress disorder
ARDS: Acute respiratory distress syndrome
HRQoL: Health-related quality of life
SF-36: Short Form Health Survey 36
PGWBI: The Psychological General Well-Being Index
PSQI: The Pittsburgh Sleep Quality Index
BPI: Brief Pain Inventory
PTSS-14: Post Traumatic Stress Syndrome 14 items
HADS: Hospital Anxiety and Depression Scale
MNA: Mini Nutritional Assessment
BIA: Bio impedance-analysis
